# Are ‘red wall’ constituencies really opposed to progressive policy? Examining the impact of materialist narratives for Universal Basic Income

**DOI:** 10.1057/s41293-022-00220-z

**Published:** 2022-10-18

**Authors:** Matthew Johnson, Elliott Johnson, Daniel Nettle

**Affiliations:** 1 Northumbria University;Lipman Building, Northumbria University, Newcastle upon Tyne, NE1 8ST, United Kingdom; 2 Northumbria University;Lipman Building, Northumbria University, Newcastle upon Tyne, NE1 8ST, United Kingdom; 3 Ecole Normale Supérieure-PSL, CNRS;29 rue d’Ulm, 75005, Paris, France

**Keywords:** Public policy, Universal Basic Income, public health, socio-economic status, adversarial collaboration, materialism

## Abstract

Universal Basic Income (UBI) is often presented as desirable in theory, but unsaleable electorally. Policymakers fear intuitive, ‘values’-based opposition from socially conservative voters, whom the policy would benefit materially, but who might regard it as ‘giving others something for nothing’. We provide evidence from ‘red wall’ constituencies in Wales and the Midlands and North of England that indicates this presumption of voters is wrong. In Study 1, we find high levels of support for the policy, with different narrative framings more effective for different groups based on their material interests. In Study 2, we used a novel ‘adversarial collaboration’ method to show that simple narratives can strongly increase support for UBI even among respondents who initially see themselves as fundamentally opposed. The generated narratives stressed positive, material consequences of introducing UBI, rather than conformity with abstract values. This indicates that policymakers should exercise caution over ‘values’-based explanations for preferences.

## Introduction

Forms of Universal Basic Income (UBI) have been advanced over several decades by policymakers from across the political spectrum. Providing individuals with a largely unconditional means of satisfying basic needs has been presented as a means of dealing with labour market insecurity, bureaucratic stagnation (Gordon, 2014) regional inequality, absolute poverty and, more recently, public health (Johnson, et al., 2021). The universality of UBI as a policy has increased in salience during the pandemic with programmes such as ‘furlough’, the Coronavirus Job Retention Scheme (HM Revenue and Customs, 2021), and temporary freezes on evictions (Ministry of Housing, Communities and Local Government, 2021) being deployed to prevent destitution among non-welfare recipient cohorts. We have shown that the pandemic has increased UK public support for UBI above an already high benchmark by virtue of efficiency in providing social security and thereby reducing stress and anxiety (Nettle et al. 2021). We expect the cost-of-living crisis to increase that support further by affecting large portions of the population.

These concerns have affected political discourse, with the Work and Pensions Secretary overturning decades of Conservative ideological orthodoxy by claiming that there is no evidence that an uplift in Universal Credit payments reduces willingness to work (Butler, 2021). However, once the uplift was ended, ministers focused on the need to incentivise work (Chakelian, 2021). These moves, along with dismissal by Labour Party Shadow Cabinet members of policies such as UBI (Bloom, 2022; Rodgers, 2020; Bloom, 2021) may reflect a misreading a public mood in which only 12% of voters wish to return to the ‘old normal’ (Britain Thinks, 2020: 35). Indeed, the Welsh Government has committed to a trial of basic income for Care Leavers to commence in July 2022, with a monthly payment set above the Living Wage at £1,600 per month.

However, policymakers often respond that, although UBI sounds good in theory, it is bad in practice because it is electorally unsaleable. Politicians and commentators, even if nominally supportive, fear that advocating UBI will subtract credibility (Rentoul, 2020), offering opponents opportunities to present their politics as frivolous or incompetent (Smith, 2020). That said, numerous UK councils (Halliday, 2019) and centre-left political parties (Labour Party, 2019b: 60; SNP: Crerar, 2020) have committed to support UBI trials. This support, however, is tentative, as the change in perspective between the previous and current Labour leadership on UBI demonstrates.

One reason for caution in Labour is attribution of Conservative electoral success in former Labour heartlands in Wales and the Midlands and North of England (see MacKinnon, 2020) – the so-called ‘red wall’ – to a values-based rejection of progressive policy (Goes, 2021; Nandy, 2020). This has been interpreted by many commentators (e.g., Mason, 2021), and, it seems, by the Labour leadership (Berry, 2021), to a conflict of values within the country, with a Brexit-supporting socially conservative ‘red wall’ actively opposed to progressive redistributive socioeconomic policy. Indeed, Labour Party Leader Keir Starmer recently resurrected Conservative Party rhetoric from the 2017 General Election to claim that there would be ‘no magic money tree economics’ from him and Shadow Chancellor Rachel Reeves (Crerar & Buchan, 2022).

This ideational understanding of preferences suggests that parties ought to tailor policies to values as fixed social phenomena. Given the pivotal role of ‘red wall’ constituencies in UK elections, policymakers have professed what we term an ‘insurmountable conservative values thesis’ – that endorsing radical socioeconomic policies is necessarily self-defeating for parties, even when those policies necessarily benefit target voters. However, materialist accounts have long suggested that people’s preferences are formed by their material circumstances and the strategies deployed to advance their perceived interests (see Maguire, 2020). Analysis of Brexit voting patterns suggest little cultural variation within England, with income and age as key determinants of voting patterns (see Goodwin and Heath 2016; Stark, 2017). Given the recognised importance of ‘levelling up’ and addressing inequality, it is necessary to test the ‘insurmountable conservative values thesis’ by presenting voters with arguments for UBI that outline pathways to enhancement of material interests. In this article, we use survey data to show that deploying these methods can elicit high levels of support for UBI among ‘left-behind’, ‘red wall’ voters. Moreover, we present evidence from adversarial collaboration to indicate that even those who are initially sceptical become much more supportive of the policy when ways in which it could advance their material interests are presented to them in simple needs-based arguments.

### What do we know about people’s appraisal of policies?

In recent years, there has been a conflict between idealist analyses of policy preferences, which suggest a cleavage of values, and materialist analyses, which point toward a fracturing of society along socio-economic lines. Although UBI is explicitly an economic policy, understandings of the reasons for people’s evaluation of the policy often focus on abstract values (see Hamilton, et al., 2021). In such instances, fairness is presented as a multiply realisable value inferred from statements like ‘people shouldn’t get money for nothing’. As a radical revision to existing needs- and means-based welfare systems, UBI has often been regarded as having sat outside the ‘Overton Window’ of policies acceptable to the electorate (see Editorial Board, 2020; Cowan, 2020;; Gopal and Issa, 2021). Idealist understandings of the window present its parameters as situated within the centre of an ideological continuum and subject to discursive repositioning within the centre of that line. Associated values-based strategies attempt to present politicians as sharing values of voters as means of illustrating competence to govern within existing structures. This often leads to a preference for deontic, abstract discussion of policies in ways that exclude material consequences as immature or irrelevant (see Parker & Payne, 2021).

In contrast, materialist analyses view the window as being shaped first by the ways in which people satisfy their needs and second by the ideological superstructure that provides argumentative justification for people’s socio-economic behaviour (see Marx, 1873). The latter suggest far greater scope for radical revision of acceptability, with shifts in socioeconomic circumstance exposing differences in material interests and presenting opportunities for new means of meeting enduring human needs. Materialist strategies focus on consequentialist justification, viewing values as multiply-realisable narrative devices. The values-based position is inherently conservative and fosters concern for symbolism in search of a putative centre; the materialist position entails presenting the consequences of policies, the propensity those policies have for meeting people’s needs in the current circumstances of society.

The salience of materialist accounts is supported within behavioural science. Nettle & Saxe (2020) have found that people’s political and institutional preferences are shaped by perceived circumstances, with evaluation of policies on inequality and distribution of resources dependent on perception of luck (see also Piff et al., 2020), threats (such as conflict), and availability of resources. Recently, we (Nettle et al., 2021) demonstrated that support for UBI had risen in response to people’s recognition of increased exposure to risk of destitution and heightened need for efficient means of mitigating that risk. While particular pandemic or conflict-related crisis may come and go, crises of capitalism and associated economic policies show no sign of being resolved.

The long-term effects of deindustrialisation and the rolling back of the state from the 1970s onwards has contributed to increasing regional and geographical inequality, with large parts of England and Wales outside the South East ‘left behind’ (Rice & Venables, 2021). That process was exacerbated by the Global Financial Crisis, which forced many small- and medium-sized enterprises to close, reducing local opportunities for social mobility (see Corrado & Corrado, 2011). This was then compounded by a decade of austerity that reduced welfare spending and public employment further (Macleod & Jones, 2018). That left communities, and the UK as a state, vulnerable to the COVID-19 pandemic and with expanding inequalities. While commentators have explained the Brexit vote and the Conservative victory at the 2019 General Election that was defined by a campaign centred around ‘getting Brexit done’ as being rooted in conflicts of values, there is a body of evidence to suggest that people’s preferences were grounded in recognition of divergent material interests (see Stark, 2017) as those ‘who have been ‘left behind’ by rapid economic change and feel cut adrift from the mainstream consensus were the most likely to support Brexit’. Indeed, the gap between support for leave among graduates and those with GCSEs in low-skilled areas was half that in high-skilled areas (Goodwin and Heath 2016). Even before the COVID-19 pandemic, support for tax and spending and belief in the legitimacy of state involvement in the economy had risen to medium-term highs (see NatCen, 2018). The pandemic has only heightened that consensus, with both the UK Conservative Government and the US Democratic Administration believing that the state alone has the capacity to address myriad challenges associated with regional inequalities, health, housing, infrastructure and climate (see Johnson, Flinders and Degerman., 2021). This represents a significant shift in public perception and policy development that cannot easily be explained through reference to values.

Just as nominally centre right parties have adopted commitments to once unthinkable state investments in the economy (see Levy, 2021), the UK Labour Party has eschewed its more radical recent policy commitments. Its current leadership has attributed its loss in 2019 to the clash of radical policy manifesto commitments, such as UBI, with the values of former voters in ‘red wall’ seats who swung Conservative. What we describe as the ‘insurmountable conservative values thesis’ suggests that voters’ natural conservatism mean that it is self-defeating to present progressive policies, such as UBI, that have the capacity radically to enhance voters material interests (Peat, 2020). UBI is an exemplar of such policies insofar as it calls for radical changes to existing welfare and fiscal systems. As such, it is an excellent test case for the thesis with specific relevance to a critical constituency within the UK, but also more broadly in other democracies, such as the US, in which ‘left behind’ communities are regarded as naturally opposed to progressive change.

Evidence on public perception of UBI is mixed. In the European Social Survey of 2016, 56% supported the idea of UBI, with the proportion exceeding 45% in 20 of the 23 countries (Roosma & van Oorschot, 2019), while majorities of people find pro-UBI arguments based on provision of security and reduction of bureaucracy convincing (Ipsos MORI, 2017). However, support reduces when respondents are told that a UBI scheme would come at the expense of higher taxation or reducing existing welfare benefits (Ipsos MORI, 2017). Pluralities of respondents feeling that the money could be better spent targeting help to the poor (Young, 2018) or believing that there would be negative consequences for willingness to work (Dalia Research, 2017). There is also the issue of cost, which we set aside here but have calculated could be met through a combination of progressive fiscal reform and returns from reduced burdens on healthcare systems, reduced sickness, reduced crime and increased productivity (Reed et al., 2022). However, even where evidence presents universality as a source of disapproval, there is also evidence to suggest that narratives can increase support (Rincon, 2021),

The behavioural science and experimental economics literatures present several concepts by which to categorise people’s non-cost-based concerns about UBI. First, there is concern for the distinction between relative and absolute gains. Even if all, or nearly all, the population would gain from the introduction of UBI (Reed et al. 2022), some groups would gain more than others. People are not exclusively concerned with their absolute gain, but may also wish to prevent others gaining disproportionately (Gil-White, 2004: 272). UBI has often been framed as a measure to benefit the poor above all, and most voters do not regard themselves as poor. Thus, the policy is assumed to be of disproportionate relative benefit to others (see Hamilton et al., 2021).

Second, there is a deservingness heuristic: help should go only to those in need, recipients should do something in return, and recipients ought not to be responsible for the need that has befallen them (see Nettle & Saxe, 2020). As a universal benefit, UBI dissolves the distinction between ‘deserving’ and ‘undeserving’ poverty. Third, there is concern for flourishing (eudaimonia), such that people should not be, or encouraged to be, inactive, because it is harmful to theirs and others’ wellbeing (see Johnson, 2013). This historical, Aristotelian account of flourishing is materialist insofar as it regards materials and their effective deployment as being central to achievement of tangible goods. UBI may be seen to reward or foster fecklessness in misusing material resources. Fourth, there is concern for interdependence, such that society depends upon reciprocity in order to function. UBI may remove the everyday ties of reciprocity forged through employment, leaving individuals unwilling to contribute to society (see Gilbert et al., 2018). Fifth, there is concern that UBI might enable the state to dominate individuals by imposing dependence (see Lazar, 2021).

To test the insurmountable conservative values thesis, we engaged with these concepts to frame materialist narratives for presentation to ‘Left-behind’ voters.

### Framing UBI for the public

In examining human persuasion, Walter Fisher (1987) contrasts two paradigms. In a rational choice paradigm, actors develop preferences by evaluating arguments according to the quality of evidence presented. In the narrative paradigm, human beings are storytellers with pre-existing beliefs who identify what they see as good reasons for preferences from historically and culturally contingent stories. Proponents exist for each paradigm, with Morgan et al. (2002) contending that narrative is more persuasive than statistics, and Hornikx (2007) asserting the opposite. On UBI, Greve (2020) highlights evidence of impact from narratives on perceptions of welfare policy via the perpetuation of ‘myths’ that link people’s often genuine concerns for their material interests to often self-defeating policy responses. This is apparent in the opposition of many of the poorest in society to taxing the richest (Shapiro, 2002) or with support for inequalities justified through skill and opportunity in the ‘American Dream’ being negatively correlated with higher socio-economic status (SES) (Hochschild, 1995). Shapiro (2002: 122-123) argues that ‘downward framing effects’ that present the poorest as a threat are particularly effective because individuals have a tendency to underestimate inequality and overestimate their position within social hierarchies, meaning that they seldom associate themselves with the poor and oppose measures that would actively enhance their own incomes.

However, there is evidence that narratives can be deployed successfully to highlight people’s genuine material interests, for example regarding shifting perceptions toward egalitarian socio-economic policies (Piff et al., 2020). Where narratives are successful, they ‘help each other see from different perspectives’ (Stone, 2011: x), invoking interests in ways that align with the content of policies.

Those on the left, who are more likely to support UBI, have often focused simply on highlighting points of conflict (Katz, 2010: 489) without creating a tangible story of redemption. The consequence is policy perceived as being concerned for the interests of others, without satisfying desire for aspiration within the population (see Fielding, 2016). Jeremy Corbyn’s pledge to eliminate homelessness (Labour, 2019a) would have addressed a compelling need, but could be regarded as relevant only as a moral act toward ‘others’. Current framings of UBI, exacerbated by existing trials, as a benefit for those out of work foster similar perceptions. This is self-defeating, since, under most funding formulations, UBI would constitute a gain in real terms for most recipients (Lansley & Reed, 2019) and would benefit specifically those who do not currently receive benefits. What, then might a narrative for promoting UBI look like?

The UK is deeply unequal in both wealth and health. Home ownership and wealth are disproportionately concentrated in older age groups (though the correlation is not linear) (Arundel, 2017) who are also more likely to have poorer health. Older age groups are most likely to endorse the deservingness heuristic (Goodwin & Heath, 2020). Precarious work (Papoutsaki et al., 2019: 7) and renting are disproportionately concentrated in younger age groups (Tinson & Clair, 2020) who are less vulnerable to ill health by virtue of age. Given the apparent distinction in material interests between those who are younger and less likely to own property and those who are older and more likely to own property, there is *prima facie* reason to suppose that different narratives are required for different sections of the electorate.

One narrative ‘principle’ is to shift emphasis from concern with poverty and unemployment, to support for the aspirations of those in-work. While UBI may benefit the former by redistributing wealth and removing conditionality, our work (Johnson et al., 2022) suggests that there are key benefits to the latter. For example, there is the potential for reducing stress-related ill health and improving the wellbeing of workers (Johnson et al., 2019) as well as providing opportunities to upskill and advance business (D’Mello, 2019). A second principle results from the fact that the pandemic has enabled a much broader section of society to foresee the possibility of destitution (Nettle et al., 2021) for reasons beyond their control. It may be possible to frame UBI in terms of relative gains for those striving in work by contrasting it with a needs-based system that rewards others at their expense (see Johnson and Nettle, 2020).

A third principle is to emphasise health. The pandemic has demonstrated the interdependence of individuals’ health and increased awareness of social determinants and the national impact of health inequalities (Patel & Kariel, 2021). The Marmot Review (Marmot et al., 2010: 19) found that, in England, 1.3-2.5 million extra years of life and 2.8 million years free of illness or disability were being lost annually due to health inequalities. Worsening health inequalities (Marmot et al., 2020: 149) cannot be addressed by focusing solely on the poorest (Marmot et al.,, 2010: 16). The Government’s ‘prevention agenda’ (Department of Health and Social Care, 2018) supports an emerging consensus that prevention of ill-health can only be achieved by addressing such social determinants as poverty and inequality (The Lancet, 2020). We have elsewhere suggested that UBI could serve as an effective upstream intervention (Johnson et al., 2022). During the pandemic, in-work poverty has rendered some individuals more likely to contract and spread the disease by virtue of their need to work even while ill (Whitehead et al., 2021). The interests of healthy, otherwise comfortable, workers as well as retired people are intimately bound up with the need of others *not* to work under conditions conducive to contagion and *not* to work while ill.

Given that there is a divergence in material interest between older people who are more likely to own their own homes but are vulnerable to disease, and younger people who are less likely to own their own homes but also less vulnerable to disease, there is *prima facie* reason to expect differential effectiveness of the two major narrative framings, the economic and the health-based, for different groups of voters. However, both those framings emphasise positive consequences to people’s ability to sustain life.

### The present research

Our data come from two related studies conducted during May-September 2021. In Study 1, we surveyed residents of 42 ‘red wall’ constituencies (n = 858) to establish baseline levels of support for UBI, to understand the demographic predictors of this support, and to examine the relative effectiveness of two narrative framings that appeal to people’s interests. One framing is based on economic benefits, and another on health benefits. We found high levels of support overall, and, that the effectiveness of the two framings varies in ways that relate to people’s material interests. The economic narrative is more appealing to non-homeowners, whereas the health narrative is more appealing to homeowners, whose material circumstances are more secure but are nonetheless subject to the vagaries of possible ill health.

In Study 2, we used a novel ‘adversarial collaboration’ approach to generate narratives that addressed people’s reservations about UBI. We invited a small number of participants (n = 20) who had expressed fundamental opposition to UBI to nonetheless design narratives that would persuade people like them of the merits of the policy. The narratives our respondents generated were strongly focused on the potential positive consequences of UBI for human needs, rather than conformity with abstract values. We condensed these narratives into six synthesised versions, each organised around a single positive consequence of UBI. We then presented the six synthesised narratives to other ‘red wall’ respondents (n = 105) who had previously expressed fundamental opposition to UBI. The six narratives were rated as persuasive to varying degrees, and exposure to all of them increased UBI support quite markedly. The resulting analysis is among the most comprehensive exploration of people’s evaluations of UBI conducted thus far, and also shows how this support can be shaped by alternate framings of the potential benefits of the policy. It is intended to present progressive policymakers with empirical means of shaping UBI – and, potentially, other welfare reforms – to persuade those who stand to gain most from its implementation.

#### Study 1

Study 1 focused on strength of support or opposition for UBI, which was introduced via one or the other of two narratives, an economic and a health narrative. Studies 1 and 2 were approved by the Faculty of Medical Sciences ethics committee, Newcastle University.

## Methods

### Participants

We obtained 858 responses from 42 ‘red wall’ constituencies in the North of England between 12 and 26 June 2021 via prolific.co, a crowd-sourcing platform for psychological and social research after the May Hartlepool by-election and at the height of the vaccination campaign. We followed Steve Rayson’s (2020) definition of ‘red wall’ constituencies as those historically held by the Labour Party for many tens of years – with brief exceptions in the cases of Redcar and Burnley – but were lost to the Conservatives in 2017 or 2019. We also included Batley & Spen as there was an upcoming by-election at the time which was believed to be another potential loss to the Conservatives. Rayson argues that these are seats in which there ‘were cultural barriers to voting Conservative’, ‘which voted to Leave in the EU referendum, and which have been identified as culturally conservative’ (Rayson 2020). In prolific.co, the ‘red wall’ constituencies were identified by the first part of postcodes, which means that a small number of participants may have lived just outside of relevant constituencies. A full list of constituencies and postcodes used is available at https://doi.org/10.17605/OSF.IO/2N37U. Participants were paid £1.25 for completing the survey. Mean age was 38 years (s.d. 13).

Relative to voting in these constituencies at the 2019 General Election, our sample over-represented people who voted as compared to not voting, over-represented Labour voters and under-represented Conservative voters (table S1). In the statistical analyses that follow, we have therefore applied post-stratification weights that make our sample representative of the constituencies with respect to 2019 voting behaviour.

### Design and measures

Our materials and analysis plan were pre-registered and are available at https://doi.org/10.17605/OSF.IO/2N37U.The survey first described a UBI as ‘a system in which every adult British citizen would be given a payment each month that meets your basic needs. Unlike current welfare, it is not affected by whether you work or how much money you have. Its supporters come from across the political spectrum’. Participants were then presented with one of two narratives of similar length with similar reference to the pandemic as a context, but one focusing on health benefits and one on economic (for narratives, see Supporting Information). After reading, respondents were asked to rate their level of *support for UBI* via a horizontal slider anchored with 0=strongly disagree and 100=strongly agree.

In part 2, respondents were asked whether they had heard of UBI previously and, if they had, to describe their reasons for supporting or opposing it prior to the survey in a free text box. We then asked them to rate how much the information in the survey had increased or decreased their support for UBI (henceforth *impact of narrative*), with responses via another horizontal agreement slider. The survey then solicited via a free text box a description of any factors that could make respondents more likely to support the policy.

Finally, in part 3, respondents were asked to provide basic demographic information (age, gender, socioeconomic status [SES]) plus left-right political orientation via a horizontal slider anchored with 0=far left and 100=far right, regularity of previous voting participation, the party voted for (if any) in the 2019 General Election, and which party participants intend to support in the next General Election.

### Data analysis

Data were analysed in R (R Core Development Team, 2018). Weighted proportions were obtained using R package ‘weights’. Data were weighted to reflect preferences at the 2019 General Election in order to eliminate sampling bias in favour of Labour voters. Inferential analyses used weighted general linear models, as outlined in the Results section. Categorical variables were contrast coded, and continuous variables scaled. The distribution of residuals for all models was satisfactory. All p-values are two-sided. Our main pre-registered confirmatory prediction was that older homeowners would be more likely to support UBI when presented with narratives that emphasise health benefit and younger non-homeowners when presented with narratives that emphasise economic benefit. The rest of the analyses are considered exploratory. Raw data and R scripts are freely available at https://doi.org/10.17605/OSF.IO/2N37U.

## Results

### Overall support for UBI and receptivity to arguments

The overall level of support for UBI was high, regardless of treatment (mean 71.99, s.d. 26.45). A large proportion of respondents expressed strong support (≥70, 64.9%), including 22.7% who assigned the maximum of 100. A small proportion expressed strong opposition (≤30, 10.5%). Respondents mostly indicated that the impact of the narratives on their support was positive (61.7%), or had no effect (30.0%), with just 8.3% indicating a negative effect. Respondents who had not heard of UBI previously indicated a more positive impact than respondents who had (heard: 61.2, s.d. 22.2; not heard: 72.0, s.d. 22.0; t = 6.43, p < 0.001).

### Treatment effects

There was no evidence of a treatment effect on support for UBI overall (health treatment: 72.55, s.d. 25.98; economic treatment 71.42, s.d. 26.94; t = 0.62, p = 0.53). Likewise, there was no treatment difference in the rated impact of the narratives (health treatment: 66.45, s.d. 22.34; economic treatment 64.78, s.d. 22.87; t = 1.07, p = 0.28).

### Predicting levels of support by age and home ownership

**Figure 1:**
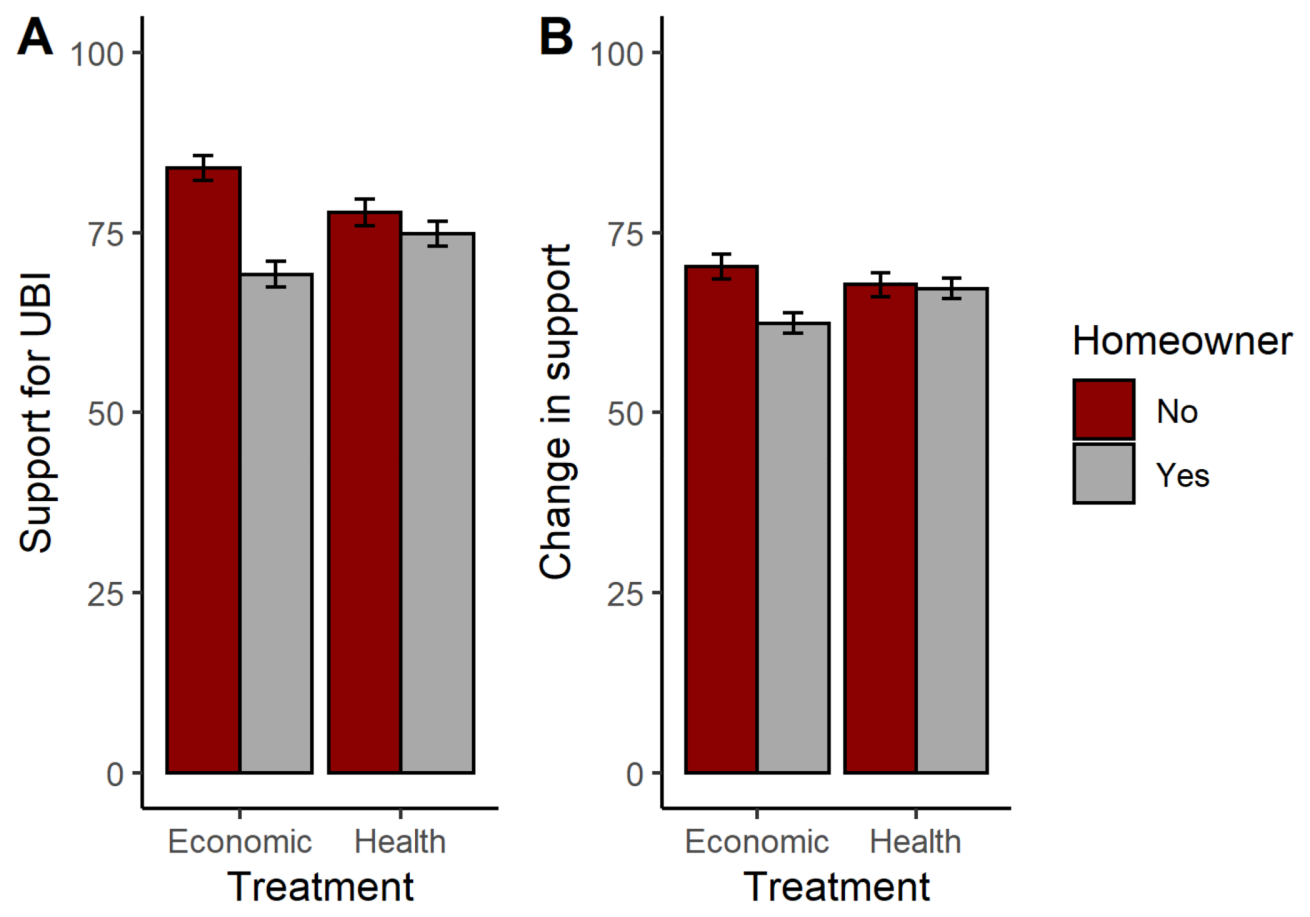
A. Mean support for UBI (± one standard error) by homeownership and narrative treatment, Study 1. B. Mean impact of narratives (± one standard error) by homeownership and narrative treatment, Study 1.

We fitted a general linear model predicting support for UBI from narrative treatment (health vs. economic), age, homeownership, and political orientation. We included interaction terms between treatment and other variables. Results are shown in table 1. There was a significant overall effect of homeownership, with homeowners less supportive than non-homeowners, and also an interaction between homeownership and treatment. As figure 1A shows, the health narrative produced higher support among homeowners than the economic narrative did, whilst the opposite pattern held for non-homeowners. In addition to the effects of homeownership, there was a significant effect of political orientation, with people who identified as more right-wing less supportive. Neither the main effect of age, nor its interaction with treatment, were significant predictors when homeownership was included in the model.

We repeated the general linear model but with impact of the narratives as the outcome variable. This produced a similar pattern (table 2; figure 1b): less positive impact on homeowners than non-homeowners, an interaction (marginally non-significant in this case) between treatment and homeownership; and less positive impact on more right-wing people. The pattern of the marginally non-significant interaction was the same, with the health narrative to be more effective than the economic narrative among homeowners, and the reverse pattern for non-homeowners.

## Discussion

We found notably high levels of support for UBI in the ‘red wall’ constituencies, both in terms of the proportion of individuals whose support was above the mid-point, and how high the level of support was on the quantitative scale. Unweighted, mean support was 75 on a 100-point scale. This equals the level in our September 2020 national surveys (Nettle et al., 2021). Although small, we explained the reduction in support in that sample compared to surveys conducted in April 2020 (mean 80) through reference to reduction in ‘the shock of the threat’ due to the pandemic. The present Study 1 was conducted during the roll out of the SARS-CoV-2 vaccine at a time when pandemic restrictions were being reduced. As such, the high level of support seems unlikely to be a very brief pandemic-related spike. Moreover, the overall levels of support were high regardless of which narrative framing, economic or health-related, we used to introduce the policy.

The widespread and strong support for UBI in these constituencies may reflect the particular conditions in which people within the ‘red wall’ find themselves. As ‘left-behind’ communities, in a country with radical levels of regional and geographic inequality, their material circumstances (income, wealth, etc.) and outcomes (health, education, etc.) are significantly below the national average. This means that the risk of serious illness and destitution overall is greater than in our previous samples, which were not nationally representative. A materialist explanation is more plausible, in this context, than an idealist one, given the degree to which such communities are often regarded as being socially conservative and intuitively opposed to ‘free money’. The high level of support for UBI that we observed stands that assumption on its head.

Confidence in a materialist explanation is further increased by the significance of homeownership for support for UBI in general, and for which narrative framing was more effective. Homeownership is a significant constituent part of the material circumstances affecting people’s preferences. Owning a home provides a degree of security in the satisfaction of needs that is not found in renting or sharing accommodation with family or friends. As such, it makes sense for homeowners to be less in favour of UBI as an economic intervention, since their material security is greater. On the other hand, they should still be amenable to health-based narratives than economic ones, since these provide added value beyond the material security granted by their existing wealth. Conversely, renters, by virtue of their lower levels of security, are more likely to value economic narratives as a precondition of other goods, including health.

Although the data showed high levels of overall support across both treatments, we also found a small number of respondents firmly opposed to UBI. Given that it is these opponents whose views are invoked by policymakers as justification not to advance UBI, in Study 2, we sought (a) to understand the reasons for their opposition, even in light of the additional narratives above, and (b) to investigate how biddable in the light of better arguments and narratives that opposition was. For this part, we used a novel form of adversarial collaboration, where we asked the participants who most opposed the policy to generate those arguments and narratives for us.

### Study 2

Study 2 took place between July and August 2021 and focused specifically on understanding the preferences of ‘fundamentalist’ opponents of UBI within ‘red wall’ constituencies in Wales and the North and Midlands of England. The study was grounded in adversarial collaboration (Tetlock & Mitchell, 2009), which is an emerging method in the natural and behavioural sciences. It is intended to improve research via collaboration with those who oppose a method or finding, and partly involves each side exploring what it would take to convince them of the position they currently oppose. It has rarely been deployed within the social sciences and not, to our knowledge, in examination of public preferences in public health policymaking with regard to ‘upstream’ interventions. As such, this represents a significant advancement of both the method and understanding of preferences within political science. In a first stage, we asked a subset of respondents from Study 1 who had stated strong opposition to UBI to generate the best arguments they could in the policy’s favour. In a second stage, we then tested how persuasive those arguments were, and how effective at increasing support for UBI, in a separate sample of strongly opposed respondents from similar populations.

## Methods

### Participants

In the first stage, we recontacted 20 respondents to Study 1 who were fundamentally opposed to UBI (≤30 level of support). They participated between 20 and 26 July 2021 via prolific.co in return for an additional £10. For the second stage, we conducted a screening survey of 677 prolific.co members from ‘red wall’ constituencies in Wales and the Midlands of England who were paid £0.25 each. We then contacted those who were fundamentally opposed, in addition to the remaining fundamentally opposed participants from Study 1 who had not taken part in the first stage. This produced a total of 105 fundamental opponents, who participated between 26 August and 2 September 2021 in return for £4.00.

### Design and measures

Our materials and analysis plan were pre-registered and are available at https://doi.org/10.17605/OSF.IO/2N37U. We first examined the free text reasons for opposition to UBI from the Study 1 data of fundamentally-opposed respondents. As we were interested in in-principle rather than pragmatic objections, we did not select respondents whose opposition was motivated by either concerns about the cost of a UBI scheme, or the fact that there is not as yet much evidence. This left a range of moral, instrumental or tautological (‘I object because I object’) bases for opposition.

We invited 20 of the remaining opponents to produce narratives in favour of UBI that would appeal to people like them. Participants were asked to spend 15 minutes producing narratives that they felt could persuade their friends and family. The narratives generated tended to discuss one or more positive consequences of UBI for people in society meeting their material and other needs. From the raw narratives, we created six ‘synthesised’ narratives, using original wording where possible (see Supporting Information). Each narrative made multiple points, but they had different central emphases. For heuristic reporting reasons, we allocated conceptual grouping terms to each narrative, but did not present these to the participants. The emphases were: 1) ‘UBI would deal with the economic crisis’ (henceforth ‘economic crisis’); 2) ‘Introducing UBI would produce evidence’ (‘evidence’); 3) ‘UBI would most help those who work hard’ (‘relative gains’); 4) ‘UBI would help people flourish in their material lives’ (‘flourishing’); 5) UBI is more efficient (‘efficiency’), and 6) ‘UBI would provide security (‘security’). Each narrative was 160-180 words. Participants were presented only with the narratives, not the grouping terms.

For stage 2, the screening survey presented participants with the initial definition of UBI used in Study 1 and asked them to rate their opposition or support on a scale of 0-100. In addition, we asked about age, home ownership and voting, plus items on perceived control over their lives, and perceived risk of destitution (100-point scales). After the screening, we approached all participants who rated UBI ≤30 (plus the remaining Study 1 participants with ratings ≤30), reminded them of the basic definition of UBI, then presented them with all six synthesised narratives in randomised order. We asked them to rate the narratives according to persuasiveness using a horizontal slider anchored with 0=extremely unpersuasive and 100=extremely persuasive. Finally, we asked them to now state their support for UBI, and the extent to which their views had been affected by what they had read (both 100-point sliders).

### Data analysis

Data were analysed in R (R Core Development Team, 2018), using linear mixed models to account for the multiple responses from each participant. Our confirmatory predictions were that those who strongly rejected UBI would be homeowners and have voted Conservative in 2019; that people who are fundamentalist opponents of UBI would produce narratives that can persuade similar individuals to support the policy, and that narratives have a different effect on people based on whether they own their home. Raw data and R scripts are freely available at https://doi.org/10.17605/OSF.IO/2N37U.

## Results

### Characterisation of sample

Table S2 provides demographic and descriptive information on our sample. As expected, the ‘fundamentalists’ contained a disproportionate share of homeowners (63.8%, versus 55.8% in stage 1), and Conservative voters (46.7% versus 19% in stage 1). Participants generally reported high levels of perceived control in life (mean 67.95, s.d. 21.05), and low perceived risks of destitution (mean 24.91, s.d. 26.43).

### Persuasiveness of justifications

**Figure 2:**
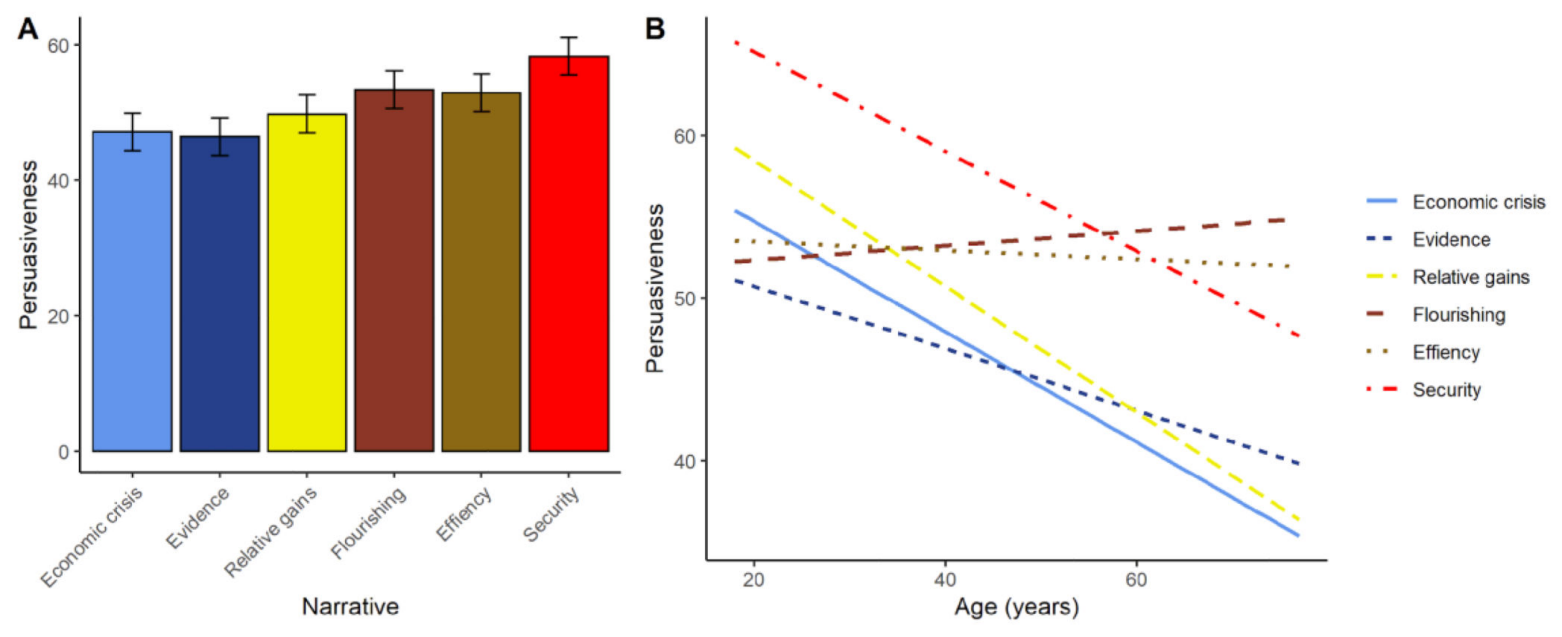
A. Estimated marginal mean (± one standard error) for persuasiveness for each of the six narratives, Study 2. B. The relationships between age and persuasiveness for each narrative, Study 2. Lines represent linear fits.

Overall, the justifications were rated, on average, around the mid-point of the scale (i.e., neither completely unpersuasive nor completely persuasive; mean 51.30, s.d. 28.74). There were significant differences in persuasiveness across the six justifications (F(5, 520) = 6.34, p < 0.001). Specifically, justifications, flourishing (t = 2.50, p = 0.01), efficiency (t = 2.31, p = 0.02) and security (t = 4.47, p < 0.001) were rated as significantly more persuasive than the least persuasive justification, economic crisis (figure 2A).

### Persuasiveness and participant characteristics

We ran a mixed model including participant age and homeownership as additional predictors, in interaction with narrative. In this model, the significant effect of narrative remained (F(5, 510) = 2.87, p = 0.01). Neither the main effect of age, nor any effects involving homeownership, were significant. However, there was a significant interaction between narrative and age (F(5, 510) = 3.22, p < 0.01), suggesting that age modifies the differences in persuasiveness across narratives. Figure 2B plots the relationship between age and persuasiveness for each narrative. As the figure shows, for economic crisis, evidence production, relative gains and security, persuasiveness declines with age. However, the persuasiveness of the flourishing and efficiency narratives show a flatter relationship with age, meaning that they become the most effective justifications specifically in the older respondents.

### Post-study support for UBI

Post-study support for UBI was dramatically higher than pre-study support (mean 46.99, s.d. 28.60, compared to pre-study mean 15.56, s.d. 10.25). This represents a mean increase of 31.43 points on the 100-point scale (t = 27.07, p < 0.001). Accordingly, participants reported that their views on UBI had been substantially affected by the arguments they had read (mean 47.85, s.d. 30.43). Moreover, the more they felt their views had been affected, the greater their increase in support (r = 0.60, p < 0.001).

## Discussion

Study 1 demonstrated that there were higher levels of support for UBI in English ‘red wall’ constituencies than policymakers might have assumed. Study 2 demonstrated that even those fundamentally opposed to a basic description of UBI are mostly amenable to persuasion by simple narratives stressing the positive consequences of UBI for people’s needs. Even a small amount of argumentation for the policy had quite a large effect. Notably, the pro-UBI narratives we generated were appropriate to sceptics of the policy in the target population. This was achieved by the adversarial collaboration strategy of getting such individuals to generate the narratives in the first place.

The narratives generated by the adversarial collaboration highlighted the value of tying material interests to people’s sense of their own capacities and self. The more successful narratives presented circumstances that respondents could identify as affecting them directly. Importantly, need was invoked in a way that respondents believed legitimate in relation to their own behaviour. For example, the successful ‘flourishing’ narrative invoked a ‘living pension’ and stressed the lack of disincentives to work hard that are inherent in UBI. It is important to emphasise that these are not abstract, values-based deontic, but tangible, materialist, outcomes-based, concerns bound up with the importance of resources to fundamental goods. Fundamentalist opponents often referred to themselves in such terms as ‘hard-working’, ‘aspirational’ and ‘independent’. Given that sense of self, and as they are disproportionately home-owning, it is possible that they conceive legitimate need as emerging from those circumstances that would substantively subtract *their* agency and leave *them* destitute – illness, injury or pernicious decisions by government. Assessing need through reference to the self is important because it necessarily depends not on abstract value but instead on a person’s material conditions and is necessarily prone to change. This reflects the findings of our previous study that demonstrated higher levels of support for UBI under pandemic conditions (Nettle et al., 2021). These findings suggest policymakers should shape narratives that highlight people’s fundamental interests, identify how the policy promotes those interests and invoke fairness as a narrative device.

The diversity of the six narratives, and their varying persuasiveness across age groups, demonstrate the need for different group-specific policy messages. Proximity to receipt of pensions and a higher rate of home ownership among older people may explain the declining levels of persuasiveness among four of the six narratives, particularly in terms of UBI as a means of dealing with an economic crisis and increasing security. On the other hand, flourishing – which emphasises impact on behaviour that may affect them and their family – and efficiency, which alludes to tax burdens that may affect them – were more persuasive for older people. It appears that the immediacy of economic insecurity means that younger cohorts have more focused concerns than older and generally more secure cohorts.

Overall, generating narratives through adversarial collaboration enabled us to understand and invoke salient interests more effectively than UBI proponents have previously been able to. We chose to synthesise the narratives in order to present justifications wrapped around core ideas, and to even out varied style in the ‘raw’ narratives. Future research might find raw narratives, despite variation in grammaticality and presentation, to be just as, or even more effective, stemming from their contextual authenticity. Alternatively, a single more heavily edited narrative that contains all main arguments in one package could be tested. Study 2 participants’ levels of support for UBI increased very substantially after having been presented with the set narratives. There may be a cumulative effect of integrating the various arguments. As we only asked for support for UBI once, at the end, we are unable to establish the cumulative or dose-dependent effect of exposure to more pro-UBI arguments.

It is important to note Chrisp, Pulkka & Garcia’s (2020, 234) finding that

In the abstract, the idea of providing a guaranteed basic income to all is popular in many countries. However, the extent of that support appears to be inversely related to the level of detail provided about the policy, particularly when the costs are clearly cited.

Our initial descriptions of UBI were at that abstract level and we deliberately set aside concerns about cost here. The studies were intended to examine other concerns and, in study 2, those who objected to UBI on the basis of a household economics understanding of money (that money is finite and cost cannot be borne) were excluded. One narrative referred to the costs of administration and loss to fraud in error under the existing system, but this was intended to convey inefficiency. These design choices should be considered limitations of the study. However, there are two salient considerations on cost that inform our analysis of findings. First, tax-benefit microsimulation modelling has demonstrated that a modest, but impactful, UBI is achievable in a fiscally neutral manner with only minor changes to the tax system (Reed et al. 2022). Second, there is strong evidence that the public’s perception of what counts as ‘expensive’ or ‘unaffordable’ – if this is even a consideration anymore – has been reshaped by the pandemic (Nettle et al., 2021), the lack of personal consequences from public debt and now the cost-of-living crisis. Voters’ loss aversion appears to have shifted from potential tax and public debt burdens to immediate threats of destitution from costs that can only be mitigated by state intervention. Sloman (2020) suggests that after ‘a long period of austerity, there are signs that the preoccupation with fiscal credibility which has prevailed since the late 1970s may be losing its force’, with Labour’s 2017 General Election result showing that ‘“big ticket” retail policies can still capture voters’ imagination, particularly if they are clearly communicated and chime with voters’ own sense of priorities.’ He concludes that despite ‘the success of the Conservatives’ attack on Corbyn in the 2019 election, the 2020s seemed likely to be a period of state expansion even before the outbreak of the COVID-19 pandemic.’ (Sloman 2020) There may also be signs that the Labour leadership under Keir Starmer has begun to recognise this. Its recently announced commitment to freezing the energy price cap at a cost of £29bn over six months (Labour Party 2022) marks a departure from an otherwise fiscally conservative approach. It reflects findings from an Opinium poll for 38 Degrees (2022) which found that 85% of 2019 Conservative voters (86% overall) support the policy. Further, in line with Gordon Brown’s proposals, 72% of Conservative voters (73% overall) back temporarily nationalising energy companies if they cannot contain bills at their current levels. Finally, 71% of Conservative voters agreed that windfall tax on energy companies and bankers’ bonuses should be used to fund extra support measures.

As with furlough policies, it appears that the proportion of the public that recognise personal exposure to risk has changed, and with that change, their support for public spending on policies to mitigate risk among a much greater proportion of the population. Despite criticisms over cost (Beckford 2022; Rogers 2022), ‘handouts’ (Bush 2022) and a perceived lack of targeting (Fitzgerald 2022) from the campaigns of candidates to be Prime Minister Liz Truss and Rishi Sunak, Labour’s price cap policy has resulted in a surge in support both for the party – which is leading in the polls at the end of August 2022 – and for Starmer in terms of who would make the best Prime Minister compared with his potential Conservative rivals (Helm 2022). This may be inferential evidence that the Conservative Party’s ‘tax bombshell’ attack line (Sloman 2020) can either be countered by reference to windfall and other non-income-tax measures or that an extended period of exposure to risk among the majority of the population and much greater public spending by the Conservatives, means that it will simply no longer be as effective a campaigning tool. This may also have an impact on Jordan, Ferguson and Haglin’s (2022) finding that negative arguments against UBI move support more than positive ones.

This does not mean that those opposed to UBI could not employ the methods indicated in this study. Further research is required to test the resilience of the positive narratives presented here against those counterarguments. In addition, heresthetic methods (see Riker 1986; Heppell 2013) of manipulating the decision-making process are likely to put supporters of UBI at a disadvantage, given that its supporters are generally outside positions of media and political control. However, the narrative approach presented here suggests scope for presenting a potentially salient policy effectively within those confines. The salience of UBI as a policy can only really be tested in general election conditions and existing evidence is likely stunted by the fact that neither the Government nor the official Opposition currently support the policy. In contrast, the policy has become much more prominent in Wales where the Labour Administration is undertaking a trial of basic income for care leavers. In the absence of that UK-wide party support, the findings above and the current economic context indicate that there is good reason to suggest that it has the ability to address a number of indisputably salient issues in the 2020s. Cost-of-living and public health crises, increasing job and financial insecurity, and environmental breakdown are all likely to require radical economic approaches as markets become increasingly unable to deal with scarcity for large sections of the population.

## Conclusion

The two studies indicate that policymakers who suggest that UBI is unappealing to target sections of the electorate are likely to be incorrect. Seats lost by Labour in the ‘red wall’ or ‘heartlands’ face some the largest challenges in Britain in terms of long-term decline in wealth, income and economic opportunity. Idealist accounts have suggested that voters in such constituencies possess fixed, socially conservative values at odds with their interests, leading them to reject radical policies that might improve circumstances. The evidence presented here confounds the ‘insurmountable conservative values thesis’, indicating an electorate that recognises the need for change and, when provided with narratives that invoke their material interests (as, potentially with Brexit – see Stark 2017), is receptive to UBI. As such, UBI is a perfectly plausible candidate for policymakers seeking to recapture voters. However, supporting elements of Rincon’s (2021) conclusions, progressive politicians need to make a case for UBI and similar redistributive policies by using narratives that fit particular material interests and are developed with the involvement of those who are fundamentally opposed. This may be true of many key ‘left behind’ constituencies within democracies, including the United States.

Beyond this, the data may also indicate a need to shift from traditional Weberian class-based categorisation of voters according to professional and educational status (see Breen 2006, 36) to much blunter concern for interests based on position in society. Given the increasing precariatisation of work as well as inter-generational differences in wealth, voting patterns and policy preferences may now have a clearer linear relationship to wealth and age than traditional categories of employment or, in future, educational levels. It may be that, in an increasingly insecure and materially distressed society, more accurate and effective policy development is achieved by examining income and wealth alone, with home ownership an important marker of the latter.

## Figures and Tables

**Table 1:** Regression results for support for UBI, Study 1

Predictor	*β*	95% CI [LL, UL]	Fit
(Intercept)	-0.05	[-0.12, 0.02]	
Treatment	0.00	[-0.07, 0.08]	
Age	-0.02	[-0.09, 0.06]	
Homeowner	-0.14**	[-0.22, -0.07]	
Left-right	-0.30**	[-0.38, -0.23]	
Treatment* Age	0.06	[-0.01, 0.14]	
Treatment* Homeowner	-0.13**	[-0.20, -0.05]	
Treatment* Left-right	-0.04	[-0.11, 0.03]	
			*R^2^* = .136** 95% CI [.09,.17]

* indicates p < .05.

** indicates p < .01.

**Table 2:** Regression results for impact of the narratives, Study 1

Predictor	*β*	95% CI [LL, UL]	Fit
(Intercept)	-0.01	[-0.08, 0.07]	
Treatment	-0.01	[-0.08, 0.06]	
Age	0.03	[-0.05, 0.10]	
Homeowner	-0.11**	[-0.19, -0.03]	
Left-right	-0.11**	[-0.19, -0.04]	
Treatment* Age	0.03	[-0.04, 0.11]	
Treatment* Homeowner	-0.08§	[-0.15, 0.00]	
Treatment* Left-right	-0.04	[-0.12, 0.03]	*R^2^* = .035** 95% CI[.01,.05]

* indicates p < .05.

** indicates p < .01.

§ indicates p = 0.06.
